# Cancer-Selective Induction of Apoptosis by Leczyme

**DOI:** 10.3389/fonc.2014.00139

**Published:** 2014-06-04

**Authors:** Takeo Tatsuta, Shigeki Sugawara, Kohta Takahashi, Yukiko Ogawa, Masahiro Hosono, Kazuo Nitta

**Affiliations:** ^1^Division of Cell Recognition Study, Institute of Molecular Biomembrane and Glycobiology, Tohoku Pharmaceutical University, Sendai, Japan; ^2^Divisions of Functional Morphology and Microbiology, Department of Pharmacy, Faculty of Pharmaceutical Science, Nagasaki International University, Sasebo, Japan

**Keywords:** apoptosis, cancer selectivity, sialic acid-binding lectin, leczyme, cytotoxic ribonuclease

## Abstract

Sialic acid-binding lectin (SBL) is a multi-functional protein that is isolated from oocytes of *Rana catesbeiana*. It has both lectin and ribonuclease (enzyme) properties, and therefore is called leczyme. We examined the anti-tumor effects of SBL and discovered that SBL has potential as a new type of anti-cancer drug. SBL causes a cancer-selective induction of apoptosis by multiple signaling pathways whereby RNA is its target. It is suggested that the mitochondrial pathway and endoplasmic reticulum stress-mediated pathway participate in SBL-induced signaling. The synergistic anti-tumor effects with other molecules, such as tumor necrosis factor-related apoptosis ligand and interferon γ, have been reported. In this study, we summarize the effects of SBL and focus on its cancer-selective apoptotic properties. In addition, we present a possible explanation for its cancer specificity.

## Introduction

Sialic acid-binding lectin (SBL), also named RC-ribonuclease (RNase), is isolated from oocytes of *Rana catesbeiana*. It is a basic protein consisting of 111 amino acids and 4 disulfide bonds (1–4). Functional studies of SBL reveal that SBL is a multi-functional protein having both lectin and RNase activities, and therefore it is now called “leczyme” (5–8). Furthermore, from studies investigating medicinal ribonucleases from frog oocytes, it has been revealed that SBL has remarkable anti-tumor effects that selectively target cancer cells (9).

Sialic acid-binding lectin was found to be an agglutinin of cancer cells because it agglutinates a large variety of cancer cells (1). Because SBL-induced agglutination is strongly inhibited by glycoproteins such as mucin but not asialomucin, and sialidase-treated cells reduce the cell agglutination induced by SBL, it is believed that the binding of SBL to glycoconjugates containing sialic acid on the cell surface contributes to the cell agglutination activity of SBL (2, 9). Selectivity for cancer cells is an important property of SBL. SBL reacts with many kinds of cancer cells, such as lung, gastric, cervical carcinoma, and leukemias, but not normal cells, such as fibroblast, lymphocytes, and erythrocytes (2). This cancer-selective agglutination activity of SBL may contribute to its cancer-selective apoptosis-inducing effects that we will discuss.

From the results of amino acid sequence analyses, it was found that SBL exhibits homology to various members of the RNase A superfamily (3, 6). SBL has conserved catalytic amino acids residues for RNase activity (one lysine and two histidines) and has pyrimidine base-specific RNase activity, which is a typical feature of the RNase A superfamily. The RNase activity of SBL is not inhibited by the human ribonuclease inhibitor (RI) (9), and this explains why SBL exerts an anti-tumor effect even though other mammalian members of the RNase A superfamily, which is tightly captured by human RI, do not show anti-tumor effects.

An anti-tumor effect of SBL was reported in both *in vitro* (P388 and L1210 cell lines) and *in vivo* (mouse ascites Sarcoma 180, Mep II, and Ehrlich cells) experiments (9). It has been considered that the SBL-induced anti-tumor effect is attributed to the cooperation of two actions; lectin activity that recognizes glycoconjugates containing sialic acid on the cell surface and RNase activity that cleaves cellular RNA. SBL shows cytotoxic effects in a large variety of cancer cells including P-glycoprotein overexpressed multi-drug resistant (MDR) cells (10, 11). Replicative DNA is the main target of traditional anti-cancer agents. The development of these reagents makes a great contribution to the progression of cancer therapy; however, side effects stemming from their low selectivity and resistance to these reagents are still significant problems. Because SBL exerts cancer-selective anti-tumor effects regardless of P-glycoprotein expression via new mechanisms that target RNA, it is expected that SBL has a potential as an alternative to conventional DNA-damaging anti-cancer drugs. In this study, we summarize the effects of SBL, and particularly focus on the cancer-selective induction of apoptosis. In addition, we propose a possible explanation for the cancer specificity of SBL.

## SBL-Induced Apoptosis

Sialic acid-binding lectin inhibits the growth of several cancer cell lines *in vivo* by abrogating solid tumor growth and ascites accumulation in mice at a non-toxic dose; therefore, prolonging the life span of tumor-bearing mice (9). It has been suggested that SBL binds to the target cell surface, internalizes into the cell by endocytosis, decomposes RNA in the cytosol, and then leads to the induction of an apoptotic signal. We recently investigated the potential mechanism of this SBL-induced cytotoxicity using human leukemia cell lines and malignant mesothelioma cell lines (10, 12). We found that SBL induces apoptosis in a caspase activation-dependent manner. SBL causes rigid mitochondrial perturbation before caspase activation; therefore, implicating an intrinsic apoptotic signaling pathway. Moreover, we reported the possible participation of endoplasmic reticulum stress in SBL-induced apoptosis (13). It was apparent that SBL causes mitochondria perturbation and endoplasmic reticulum stress independently and the mitochondrial pathway may be intensely involved in apoptosis induced by SBL.

In terms of other factors that affect SBL-induced apoptosis, we have reported that the heat shock protein (Hsp) 70 plays a role in the SBL-induced anti-tumor effect (11, 14). The reduction of Hsp70 expression results in an attenuated induction of apoptosis. Because the binding of SBL to P388 cell membrane is not affected by the decreased expression of Hsp70, we speculate that Hsp70 may interact with the SBL receptor or participate in the penetration of SBL into cells and subsequently affect the cytotoxicity of SBL.

## Sensitivity of Cells Against SBL

The anti-tumor effect of SBL has been examined in several different cancer cell lines and primary or immortalized non-malignant cells (Table 1). To date, 33 tumor and 8 normal cell lines have been determined to be sensitive or insensitive. In general, SBL suppresses the growth of various kinds of cancer cells [carcinoma (cervical, hepatocellular, oral, and breast), sarcoma, mesothelioma, leukemia (T-cell, promyelotic, and erythro), and lymphoma] but not normal cells (fibroblast, melanocytes, keratinocytes, and mesothelial cells). The sensitivities of some cancer cell lines (MCF-7, and Raji) are contradictory between reports but such contradictions may be caused by different experimental conditions and/or that MCF-7 and Raji have moderate level of sensitivity to SBL.

**Table 1:** **Cells that are reported to be sensitive or insensitive to SBL**.

Reference	Cells	Characteristics		Anti-tumor effect of SBL	Comments
Nitta et al. (9)	P388	Mouse leukemia		+	Anti-proliferative effect of SBL is diminished by sialidase treatment of cells
	P388	Mouse leukemia	Sialidase-treated	−	
	L1210	Mouse leukemia		+	
	Sarcoma 180	Mouse ascites (*in vivo* experiments)		+	SBL inhibits the tumor growth in mice
	Mep II	Mouse ascites (*in vivo* experiments)		+	
	Ehrlich	Mouse ascites (*in vivo* experiments)		+	
Nitta et al. (15)	P388	Mouse leukemia cells		+	SBL binds to RC-150 but not internalized into the cells
	RC-150	SBL-resistant P388 cell variant		−	
Liao et al. (16)	HFW	Normal human fibroblast		−	SBL inhibits cell growth specific to cancer cells
	NIH3T3	Normal mouse embryonic fibroblast		−	
	CaSki	Human cervical carcinoma		+	
	HA-22T	Human hepatocellular carcinoma		+	
	KB	Human oral carcinoma		+	
	SK-Hep-1	Human hepatocellular carcinoma		+	
	Hela	Human cervical carcinoma		+	
			Differentiation		The distinct cytotoxicity of SBL on different hepatoma cells was correlated with the differentiation extent but not the proliferation rate of the cells
Hu et al. (17)	SK-Hep-1	Human hepatocellular carcinoma	Poorly	+++	
	J5	Human hepatocellular carcinoma	Intermediately	++	
	Hep G2	Human hepatocellular carcinoma	Well	+	
	BHK-21	Normal hamster fibroblast	−	−	
Hu et al. (34)	MCF-7	Human breast carcinoma		++	Overexpression of Bcl-XL diminishes cytotoxicity of SBL
	MCF-7/Bcl-XL	Bcl-XL overexpressed MCF-7		+	
			Differentiation		Differentiation is a significant factor of the selective cytotoxicity of SBL
Wei et al. (18)	HL-60	Human promyelocytic leukemia	−	+	
	HL-60 (RA)	RA treat HL-60	Induced	−	
	HL-60 (DMSO)	DMSO treated HL-60	Induced	−	
Tang et al. (22)	HL-60	Human promyelocytic leukemia		+	SBL inhibits cell growth specific to cancer cells. SBL seems to harbor a more specific anti-cancer activity, compared with Onconase
	MCF-7	Human breast carcinoma		+	
	SK-Hep-1	Human hepatocellular carcinoma		+	
	HS-68	Normal human HS-68 foreskin fibroblast		−	
			ER		SBL induces cell death on ER-positive breast tumors but not on ER-negative breast tumors through down-regulation of ER and Bcl-2. The anti-cancer effect on SBL-treated ER-positive breast tumors is related to Bcl-2 overexpression, but not to Bcl-XL overexpression
Tseng et al. (23)	MCF-7	Human breast carcinoma cells	+	+	
	MDA-MB-231	Human breast carcinoma cells	−	−	
	ZR-75-1	Human breast carcinoma cells	+	+	
	ZR-75-30	Human breast carcinoma cells	−	−	
	MCF-7/Bcl-2	Bcl-2 overexpressed MCF-7	+	−	
	MCF-7/Bcl-XL	Bcl-XL overexpressed MCF-7	+	+	
Lee et al. (24)	BHK-21	Normal hamster fibroblast		−	SBL induces apoptosis to JEV-infected BHK-21 cells
	BHK-21 (JEV)	JEV-infected BHK-21 cells		+	
Tatsuta et al. (10)	Jurkat	Human T-cell leukemia		++	SBL shows anti-proliferative effect of various leukemia cells including MDR cells
	K562	Human erythroleukemia		+	
	K562/adr	Human P-glycoprotein-overexpressing K562 cells		+	
	U937	Human promyelocytic leukemia		+	
	Raji	Human Burkitt’s lymphoma		+	
Ogawa et al. (11)	P388	Mouse leukemia		+++	Differing sensitivities are predicted to arise from the differences in cell surface components
	K562	Human erythroleukemia		++	
	HL-60	Human promyelocytic leukemia		+	
	MCF-7	Human breast carcinoma cells		−	
	Daudi	Human Burkitt’s lymphoma		−	
	Raji	Human Burkitt’s lymphoma		−	
	NHDF	Normal human epidermal fibroblasts		−	
	NHEM	Normal human epidermal melanocytes		−	
	NHEK	Normal human keratinocytes		−	
Tatsuta et al. (12)	H28	Human malignant mesothelioma		++	SBL induces cancer-selective apoptosis against malignant mesothelioma
	Meso-1	Human malignant mesothelioma		+	
	Meso-4	Human malignant mesothelioma		+	
	Met-5A	Normal human mesothelial cells		−	

*Cells including 33 tumor cell lines and 8 primary or immortalized non-malignant cells were reported the sensitiveness for SBL. Relative intensity of anti-tumor effects was assessed with respect to each paper, and indicated as +, ++, +++, or −. RA, retinoic acid; DMSO, dimethylsulfoxide; ER, estrogen receptor; JEV, Japanese encephalitis virus. Normal cells and negative effect were emphasized with blue and pink background, respectively*.

The first report that discovered an anti-proliferative effect of SBL was performed on mouse leukemia P388 and L1210 cell lines (9). Further investigation revealed that sialidase treatment of the cells reduces the sensitivity for SBL. Subsequently, the SBL-resistant P388 cell variant RC-150 cell line was established (15). Because SBL binds to both P388 and RC-150 cells but only enters P388 cells, it is suggested that RC-150 cells have a defective internalization mechanism. These results indicate that SBL needs to bind to the cancer cell surface and internalize into cytosol for execution of its anti-tumor activity.

A few years later, Liao et al. reported that SBL inhibits the growth of several carcinoma cell lines but does not affect normal human and mouse fibroblasts (16). Hu et al. reported a phenomenon using human hepatoma cell lines that has a distinct differentiation extent (17). The distinct cytotoxicity of SBL in different hepatoma cells correlates with the differentiation extent but not the proliferation rate of the cells. Wei et al. reported a similar phenomenon that retinoic acid (RA) or dimethylsulfoxide (DMSO)-induced differentiation resulted in HL-60 cells becoming resistant to SBL (18). These results indicate that differentiation is a significant factor for the selective cytotoxicity of SBL.

Onconase (ONC) is a promising RNase for medicinal applications. ONC was isolated from oocytes of *R. pipience* and shows approximately 50% homology with SBL in the amino acid sequence (19–21). Only a few investigations have compared the effects of ONC and SBL. Tang et al. report that compared with ONC, SBL harbors a more specific anti-cancer activity because ONC is toxic to normal human HS-68 foreskin fibroblasts but SBL is not toxic in their study (22).

Tseng et al. discovered another factor that is selective in the treatment of breast carcinoma cell lines with SBL. They tested several breast cancer cell lines and revealed that SBL induces cell death on estrogen receptor (ER)-positive breast tumors but not on ER-negative breast tumors. The anti-tumor effect of SBL-treated ER-positive breast tumors is accompanied by the down-regulation of ER and Bcl-2. They also showed that Bcl-2 overexpression, but not Bcl-XL overexpression, significantly inhibits the effect (23). Furthermore, Lee et al. showed that SBL does not affect the cell survival of normal hamster fibroblast BHK-21 cells but SBL does induce apoptosis in Japanese encephalitis virus (JEV)-infected BHK-21 cells. They showed that JEV infection enhances the internalization of SBL into cells (24).

To summarize, most cancer cells are sensitive to SBL, but all normal cells tested so far are insensitive to SBL. In addition, the sensitivity does not correlate with the proliferation rate of the cells. The binding of SBL to the cell surface, internalization of SBL into the cell, expression of some kinds of molecules such as ER, Bcl-2 Hsp70, and JEV infection, are all factors that affect the cell sensitivity to SBL.

## Cancer Specificity (Predicted Possibility) and Factors That Influence the Effects of SBL

**Figure 1:**
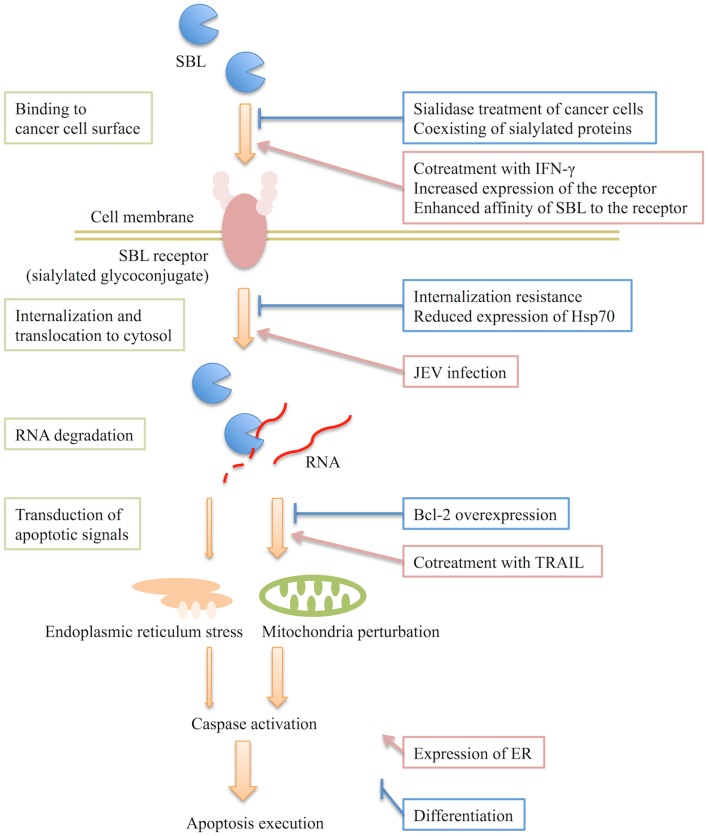
**Predicted mechanism of sialic acid-binding lectin (SBL)-induced apoptosis and proposed factors that influence the effects of SBL**. SBL binds to the cancer cell surface and then internalizes into cell. It subsequently degrades RNA in the cytosol leading to the induction of an apoptotic signal. The existence of a SBL receptor, a sialylated glycoconjugate, has been speculated. SBL induces apoptosis in a caspase activation-dependent manner. SBL causes mitochondria perturbations and also endoplasmic reticulum stress independently. The mitochondrial pathway is intensely involved in apoptosis induced by SBL. The proposed factors that influence the effects of SBL are indicated on the right and their sites of action are shown. The extent of differentiation and expression of ER are also factors, but their sites of action remain to be elucidated.

Only a paucity of information is available on how SBL specifically induces apoptosis in cancer cells. Based on the aforementioned studies, some potential mechanisms can be suggested. The proposed mechanism for SBL-induced apoptosis and the factors that influence the effects of SBL are summarized in Figure 1. SBL induces cancer-selective apoptosis via the following stages: (i) SBL binds to the cancer cell surface, (ii) SBL internalizes and translocates to the cytosol, (iii) it degrades cellular RNA, and (iv) then SBL-induced ribotoxic stress transduces an apoptotic signal via the mitochondria and endoplasmic reticulum. Factors that affect the above stages would influence the SBL-induced apoptosis. As we mentioned earlier, the binding of SBL is diminished by coexisting highly sialylated proteins such as mucin or treatment of the cells with sialidase (9). It is known that changes of glycosylation patterns on the plasma membrane occur during tumorigenesis (25, 26) and that many kinds of cancer result in an increase in the anionic content such as sialic acids on the membrane (27). Therefore, there is a possibility that an increase of anionic molecules on the cell surface may facilitate the adsorption of SBL. However, the agglutination activity of SBL is not affected by sialic acid itself (9). In addition, SBL does not agglutinate normal red blood cells even if there are sialylated molecules on the cells (2). These observations indicate that the particular structure recognized by SBL may reside in the target molecules on cancer cell membrane. Therefore, the existence of a receptor molecule for SBL has been suggested. It is possible that quantitative and qualitative changes of the receptor, such as specific or increased expression of the receptor and conformational or environmental divergence of the receptor, could affect the receptor affinity of SBL and contribute to the selectivity of SBL.

It is apparent in SBL-resistant RC-150 cells that SBL is required to internalize into the cells to show its anti-tumor effect (15). Therefore, there are factors that regulate the cell sensitivity to SBL by influencing the internalization or translocation of SBL and binding of SBL is not likely to be affected by such factors. The cytotoxic ribonuclease bovine seminal (BS)-RNase has been reported to bind both normal and malignant cells but it only demonstrates a cytotoxic effect in malignant cells. It is suggested that the differences in intracellular route for internalization contribute to the selectivity of BS-RNase (28–30). In this respect, there are several possible factors that may produce the cancer selectivity of SBL by affecting the internalization or translocation of SBL. The members of the Hsp family form a complex and interact with some receptors such as epidermal growth factor receptor (31, 32) or rota virus receptor (integrin αvβ3) (33) and regulate receptor including ligand binding and penetration. However, reducing Hsp70 expression does not affect the binding of SBL but instead attenuates its apoptosis-inducing effects. This indicates that Hsp70 participates in the intracellular routing of SBL. A JEV infection, mentioned earlier as a promoting factor, enhances the internalization of SBL (24).

Tseng et al. reported that the overexpression of Bcl-2 in a MCF-7 cell line diminishes SBL-induced apoptosis (23). We have shown that SBL causes a rigid mitochondrial perturbation during the early stages of apoptosis initiation (10). Because the antiapoptotic Bcl-2 family regulates apoptosis by retaining mitochondrial normality, it can be considered that Bcl-2 overexpression reduces SBL-induced apoptosis by affecting the mitochondria perturbation-inducing effect of SBL.

The synergistic effects of SBL with other molecules have been reported. We recently reported that SBL exerts synergistic anti-tumor effects with tumor necrosis factor-related apoptosis ligand (TRAIL) against a malignant mesothelioma cell line (12). The synergistic effects were caused by the amplification of an apoptotic signal in which Bid truncation and caspase activation resulted in drastic mitochondria perturbations (12). The combination treatment of SBL with interferon γ (IFN-γ) enhances apoptosis in MCF-7 and SK-Hep-1 cells (22, 34). Hu et al. proposed that IFN-γ somehow upregulates the expression of the SBL receptor on the cell membrane, facilitates the entry of SBL, and then results in severe cell death (34). Tang et al. found that the synergistic cytotoxicity with IFN-γ is not observed in HL-60 cells. Since SBL-induced cell death is differentiation dependent and IFN-γ is known to induce the differentiation of HL-60 cells, they suggested that this may occur because the differentiation status of HL-60 cells is altered by IFN-γ and such a change in the differentiation status protects some cell types from synergistic cytotoxicity (22).

Even if the differentiation extent and expression of ER are shown to be significant factors for cell sensitivity to SBL, the mechanisms remain to be elucidated. Low differentiated or ER-positive cells may express abundant SBL receptors or have an internalization-facilitative system for SBL. However, factors that affect SBL binding, internalization, RNA degradation, and apoptotic signal transduction are most likely the important contributors to SBL cell sensitivity. Furthermore, we assume that there are differences in the factors involved in cancer and normal cells. A comparative study that examines the SBL binding and internalization in different cancer, normal, and these-factor affected cells may help reveal the key mechanisms involved.

## Conclusion

Sialic acid-binding lectin is a leczyme that has both lectin and RNase activity. SBL elicits anti-tumor effects by degrading cellular RNA and subsequently inducing apoptosis via a mitochondria and endoplasmic reticulum stress-mediated pathway. SBL could be an innovative anti-cancer reagent because SBL induces apoptosis in cancer cells regardless of the P-glycoprotein expression and it exerts synergistic apoptosis-inducing effects with other molecules such as TRAIL and IFN-γ. SBL exhibits a high selectivity for cancer cells. Future comprehensive analyses of the SBL binding mechanisms and internalization of SBL into different cell lines will assist in determining the selectivity of SBL and help to discover new targets for cancer therapy.

## Conflict of Interest Statement

The authors declare that the research was conducted in the absence of any commercial or financial relationships that could be construed as a potential conflict of interest.
